# University-based approach to mental health services in the community

**DOI:** 10.1192/j.eurpsy.2026.10778

**Published:** 2026-07-31

**Authors:** N. V. Grinko

**Affiliations:** Clinical Psychology, Ukrainian Catholic University, Lviv, Ukraine

## Abstract

**Introduction:**

Amid Russia’s full-scale invasion, the Ukrainian people are facing pressing mental health needs. A January 2024 survey found that 77 percent of Ukrainians reported stress or severe nervousness recently (Gradus, 2024). The World Health Organization estimates that 22 percent of individuals impacted by conflict experience a mental health disorder (depression, anxiety, trauma-disorders, bipolar disorder, schizophrenia) (Charlson et al. The Lancet 2019; 394 10194). Ukraine’s mental health system is struggling to scale up quality mental health treatment to meet demands.

**Objectives:**

The Ukrainian government is actively addressing mental health needs as a result of the russian invasion and is starting to build the ecosystem needed to promote mental health in a person-centred approach.

**Methods:**

A holistic approach is essential to address these challenges, with consideration of how specific interventions will interact with each other and will explicitly fill critical gaps to ensure integration into the larger system that the government of Ukraine is currently building. A well-trained and competent mental health workforce providing care at multiple levels is essential for the delivery of effective services that enable Ukrainians to fully recover and participate in social and economic growth.

**Results:**

The role of the Ukrainian Catholic University (UCU) extends beyond academics, aiming to serve the people of Ukraine by equipping the new generation with the skills to meet modern challenges, particularly in post-war reconstruction and development. UCU’s commitment to health initiatives is reflected in various degree programs and non-degree initiatives. In addition to training, UCU Faculty of Health Sciences provides mental health services to the university and city community. Our interest is in developing and implementing a model of interdisciplinary training on mental health. At our faculty, this model could be applied to the education of physical therapy, occupational therapy, psychology and social work students. However, at master’s and postgraduate levels of education, this model can be expanded to include interaction with doctors, nurses, psychologists, social workers and other personnel necessary for mental health services.

**Conclusions:**

Over the past few years, much has been done at the legislative level in Ukraine to ensure that the ideas of interdisciplinarity can be implemented in practice, both in the education system and at the level of community services. National authorities have made considerable progress in adopting a multidisciplinary approach to mental health services, identifying specialised multidisciplinary teams as effective care providers. And the next logical step is to make this strategic approach an achievement for university interdisciplinary education as well. The developed interdisciplinary model of training and gained experience can be extended within the broader context of education and community services.

**Disclosure of Interest:**

None Declared

